# Omega-3 Polyunsaturated Fatty Acids Trigger Cell Cycle Arrest and Induce Apoptosis in Human Neuroblastoma LA-N-1 Cells 

**DOI:** 10.3390/nu7085319

**Published:** 2015-08-18

**Authors:** Wai Wing So, Wai Nam Liu, Kwok Nam Leung

**Affiliations:** Biochemistry Programme, School of Life Sciences, The Chinese University of Hong Kong, Shatin, Hong Kong, China; E-Mails: timothyso30@hotmail.com (W.W.S.); liuwainam0305@hotmail.com (W.N.L.)

**Keywords:** apoptosis, cell cycle arrest, docosahexaenoic acid, eicosapentaenoic acid, LA-N-1 cells, neuroblastoma, omega-3 polyunsaturated fatty acids

## Abstract

Omega-3 (n-3) fatty acids are dietary long-chain fatty acids with an array of health benefits. Previous research has demonstrated the growth-inhibitory effect of n-3 fatty acids on different cancer cell lines *in vitro*, yet their anti-tumor effects and underlying action mechanisms on human neuroblastoma LA-N-1 cells have not yet been reported. In this study, we showed that docosahexaenoic acid (DHA) and eicosapentaenoic acid (EPA) exhibited time- and concentration-dependent anti-proliferative effect on the human neuroblastoma LA-N-1 cells, but had minimal cytotoxicity on the normal or non-tumorigenic cells, as measured by MTT reduction assay. Mechanistic studies indicated that DHA and EPA triggered G_0_/G_1_ cell cycle arrest in LA-N-1 cells, as detected by flow cytometry, which was accompanied by a decrease in the expression of CDK2 and cyclin E proteins. Moreover, DHA and EPA could also induce apoptosis in LA-N-1 cells as revealed by an increase in DNA fragmentation, phosphatidylserine externalization and mitochondrial membrane depolarization. Up-regulation of Bax, activated caspase-3 and caspase-9 proteins, and down-regulation of Bcl-X_L_ protein, might account for the occurrence of apoptotic events. Collectively, our results suggest that the growth-inhibitory effect of DHA and EPA on LA-N-1 cells might be mediated, at least in part, via triggering of cell cycle arrest and apoptosis. Therefore, DHA and EPA are potential anti-cancer agents which might be used for the adjuvant therapy or combination therapy with the conventional anti-cancer drugs for the treatment of some forms of human neuroblastoma with minimal toxicity.

## 1. Introduction

Neuroblastoma is the second most common extracranial malignant tumor of childhood and the most common solid tumor of infancy arising from neural crest cells, which can be developed anywhere in the sympathetic nervous system [1]. In general, it accounts for around 8%–10% of all childhood cancers and about 15% of cancer deaths in children [1]. The most common site of the neuroblastoma locates at the adrenal glands and accounts for approximately 40% of the tumors. Additionally, it can also develop in nerve tissues of other sites, such as the neck, chest, abdomen or pelvis [2]. Current treatment of neuroblastoma depends on the risk categories of the patients. Those who suffer from low- and intermediate-risk neuroblastoma can be treated with surgery and low dose chemotherapy without radiotherapy, and the survival rate is satisfactory [3,4]. For high risk neuroblastoma patients, current treatment consists of intensive chemotherapy, surgery, radiotherapy, stem cell transplantation, differentiation therapy and immunotherapy [5,6]. However, the problem is not limited to the low survival rate (<40%), but also the severe and inevitable side effects seen in infants and children [7]. Therefore, there is a pressing need to develop some novel agents, especially those derived from natural products, which are more efficacious to inhibit the proliferation of cancer cells while exerting minimal cytotoxicity towards normal cells, as an alternative strategy in neuroblastoma treatment.

Omega-3 polyunsaturated fatty acids (n-3 PUFAs) refer to the long-chain fatty acids that possess their first carbon-carbon double bond at the third carbon from the methyl end [8]. Common examples of n-3 PUFAs include α-linolenic acid (ALA), docosahexaenoic acid (DHA) and eicosapentaenoic acid (EPA). ALA is found naturally in vegetable oil, while DHA and EPA are found predominantly in oily fish, such as salmons, mackerels and sardines [9]. Since ALA cannot be synthesized in our body, while DHA and EPA are barely converted from ALA, these essential n-3 PUFAs have to be taken from diet [10]. Previous reports have shown that n-3 PUFAs are required for normal function in developing tissues and appropriate maturation of a wide variety of physiological processes [11]. In addition, n-3 PUFAs are thought to have beneficial effects on human health, such as lowering the risk of cardiovascular disease and Alzheimer’s dementia, reducing serum triglyceride level, and possessing anti-inflammatory and immunomodulatory activities [12,13,14,15]. In recent years, there have been increasing studies on the anti-carcinogenic and anti-tumor activities of n-3 PUFAs. A number of epidemiologic studies have shown that intake of dietary n-3 PUFAs can exhibit preventive effects on the development of several types of cancer, including cancer of the breast, colorectum and prostate gland [16]. In addition, some *in vitro* studies have been conducted to demonstrate the anti-tumor effects of n-3 PUFAs and unravel the underlying mechanisms in different human cancer cell types. It was found that n-3 PUFAs could trigger cell cycle arrest in metastatic hepatocellular carcinoma MHCC97L cells [17] and metastatic melanoma SK-Mel-110 cells [18], and induce apoptosis in several colorectal cancer cell lines [19] and metastatic melanoma WM266-4 cells [20]. In addition to the direct anti-proliferative effect on cancer growth, n-3 PUFAs could suppress the angiogenesis process, as shown by using human umbilical vein endothelial cells [21]. Although there is increasing evidence showing that n-3 PUFAs can exert direct growth-inhibitory effect on various types of cancer cells *in vitro*, the anti-tumor effects and the action mechanisms of n-3 PUFAs on human neuroblastoma cells have not yet been investigated.

In the present study, we compared the anti-proliferative effect of three common n-3 PUFAs (ALA, DHA and EPA) on the human neuroblastoma LA-N-1 cells using the colorimetric MTT reduction assay. The results showed that the anti-tumor effects of DHA and EPA were much more potent than that of ALA, hence, they were chosen to be the specific targets for further investigation. Mechanistic studies revealed that the growth-inhibitory effect of DHA and EPA might be due to the triggering of cell cycle arrest and inducing apoptosis in LA-N-1 cells.

## 2. Materials and Methods

### 2.1. Chemicals and Reagents 

The omega-3 PUFAs (with purity ≥98%) used in the study, ALA, DHA and EPA, were purchased from Cayman Chemical (Ann Arbor, MI, USA). Stock solutions (0.02 M) were prepared by diluting the PUFAs in sterile, cell culture-tested ethanol (Sigma-Aldrich Co., St. Louis, MO, USA). All other chemicals were purchased from Sigma-Aldrich unless otherwise stated.

### 2.2. Culture of Cells 

The human neuroblastoma LA-N-1 cell is an *N-myc* gene amplified cell line established from the metastatic tumor in the bone marrow of a 2-year-old boy [22]. It was purchased from the RIKEN BioResource Center Cell Bank (Ibararki, Osaka, Japan). The cells were maintained in RPMI-1640 medium (GIBCO, Grand Island, NY, USA) supplemented with 10% fetal bovine serum (FBS; GIBCO, Grand Island, NY, USA) and 1% antibiotics (100 U/mL penicillin G, 100 μg/mL streptomycin sulfate, and 0.25 μg/mL amphotericin B or fungizone (PSF) in 0.85% saline) in a humidified incubator containing 5% CO_2_ in air at 37 °C.

The human embryonic kidney HEK-293 cells and human hepatocyte-like WRL-68 cells were purchased from the American Type Culture Collection (Manassas, VA, USA). The HEK-293 cells were maintained in Dulbecco’s Modified Eagle Medium (GIBCO, Grand Island, NY, USA) supplemented with 10% FBS and 1% antibiotics, and the WRL-68 cells were maintained in Minimum Essential Medium (GIBCO, Grand Island, NY, USA) supplemented with 10% FBS and 1% antibiotics in a humidified incubator containing 5% CO_2_ in air at 37 °C.

The primary embryonic cortical neurons from SD rats were maintained in Minimum Essential Medium supplemented with 5% FBS and 1% antibiotics, and the murine peritoneal macrophages were maintained in RPMI-1640 medium supplemented with 10% FBS and 1% antibiotics in a humidified incubator containing 5% CO_2_ in air at 37 °C.

### 2.3. Cell Growth Assay 

The MTT colorimetric assay was used to measure cell growth and viability, as described previously [23]. Briefly, LA-N-1 cells (1.2 × 10^4^ cells/well) were seeded in a flat-bottomed 96-well microtiter plate and incubated with either solvent control (0.5% ethanol) or various concentrations of n-3 PUFAs (ALA, DHA or EPA) for different periods of time. After incubation, the relative cell number was determined by the MTT assay and recorded by a BENCHMARK microplate reader (Bio-Rad Laboratories, Hercules, CA, USA). 

### 2.4. Colony Formation Assay 

Cancer cell colony-forming ability was determined as previously described [24]. The bottom of a 6-well plate was first pretreated with poly-D-lysine hydrobromide for 4 hours and then washed once with deionized water. Afterwards, LA-N-1 cells were seeded in each well (400 cells/well) and allowed to settle overnight. On the following day, cells were treated with either solvent control (0.5% ethanol) or various concentrations of DHA or EPA for 24 hours. Subsequently, the wells were washed and replaced with fresh RPMI medium. The medium was changed every 3 days. After 6 days, colonies were fixed with 100% ice-cold methanol and stained with Hemacolor staining solutions (Merck Millipore, Darmstadt, Germany). The colonies were counted under a light microscope (Carl Zeiss™ Primo Vert™ Inverted Microscope; Carl Zeiss, Oberkochen, Germany) and the percentage (%) of colonies formed was calculated as follows:

$$\text{\% of colonies formed = }\frac{\text{Number of colonies of n-3 PUFAs-treated cells}}{\text{Number of colonies of the controlcells without n-3 PUFAs treatment}}$$

### 2.5. Cell Cycle Analysis 

The cell cycle profile of the treated LA-N-1 cells was analyzed by staining the DNA content in the cells. LA-N-1 cells (1.5 × 10^5^ cells/dish) were seeded in a 60 mm dish and incubated overnight. Afterwards, the cells were synchronized with 0.5% heat-inactivated FBS overnight and incubated at 37 °C. After synchronization, the cells were treated with either solvent control (0.5% ethanol) or different concentrations of DHA or EPA for 48 hours. On the day of analysis, the cells were permeabilized with 70% ethanol at 4 °C for 30 min. Subsequently, the cells were treated with propidium iodide (PI) and analyzed by flow cytometry (FACSCanto™ flow cytometer; BD BioSciences, San Jose, CA, USA) for cell cycle distribution using the ModFit LT V3.0 software (Verity Software House, Topsham, Maine, USA).

### 2.6. Measurement of DNA Fragmentation 

The measurement was performed by Cell Death Detection ELISA^PLUS^ Kit (Roche Applied Science, Indianapolis, IN, USA) according to the manufacturer’s instruction. Briefly, LA-N-1 cells were seeded in a 96-well plate (1.2 × 10^4^ cells/well) and incubated with solvent control (0.5% ethanol) or various concentrations of DHA or EPA for 72 hours. After incubation, the cells were lysed and the supernatant was transferred into the streptavidin-coated 96-well plate. The absorbance was measured at 405 nm and the degree of apoptosis was expressed as enrichment factor, which was calculated as follows:

$$\text{Enrichment factor = }\frac{\text{Absorbance of the n-3 PUFAs-treatedcells}}{\text{Absorbance of the controlcells without n-3 PUFAs treatment}}$$

### 2.7. Analysis of Phosphatidylserine Externalization 

The phosphatidylserine (PS) externalization of the treated LA-N-1 cells was examined by Annexin V-Green Fluorescence Protein (GFP) and PI dual staining method. LA-N-1 cells were seeded in a 60-mm dish (1.5 × 10^5^ cells/dish) and treated with either solvent control (0.5% ethanol) or various concentrations of DHA or EPA for 48 hours. After incubation, the cells were resuspended in Annexin V binding buffer (BD Biosciences, San Jose, CA, USA) supplemented with Annexin V-GFP fusion protein and PI. The samples were analyzed for red fluorescence (for PI staining) with an excitation wavelength of 488 nm and an emission wavelength of 670 nm, *versus* green fluorescence (for GFP staining) with an excitation wavelength of 488 nm and an emission wavelength of 530 nm using the FACSCanto™ flow cytometer (BD BioSciences, San Jose, CA, USA). The percentages of cells at the four quadrants were calculated by the WinMDI (Version 2.9) software and the cells located at the bottom right quadrant represent the early apoptotic cells.

### 2.8. Determination of Mitochondrial Membrane Potential 

Change in mitochondrial membrane potential (Δψm) was detected by the fluorescent dye JC-1 (Molecular Probes, Invitrogen Corporation, Grand Island, NY, USA). JC-1 is capable of selectively entering the mitochondria where it forms monomers and emits green fluorescence when Δψm is relatively low. At high Δψm, JC-1 aggregates and gives red fluorescence. A decrease in red to green fluorescence ratio indicates mitochondrial membrane depolarization. LA-N-1 cells were seeded in a 60 mm dish (1.5 × 10^5^ cells/dish) and treated with either solvent control (0.5% ethanol) or various concentrations of DHA or EPA for 48 hours. Afterwards, the cells were incubated in phosphate-buffered saline (PBS) supplemented with JC-1 dye at 37 °C in dark for 30 min. The samples were then analyzed for red fluorescence (FL-2) with an excitation wavelength of 488 nm and an emission wavelength of 585 nm *versus* green fluorescence (FL-1) with an excitation wavelength of 488 nm and an emission wavelength of 530 nm using the FACSCanto™ flow cytometer (BD BioSciences, San Jose, CA, USA). The percentages of cells with membrane depolarization were calculated by the WinMDI (Version 2.9) software.

### 2.9. Protein Expression Analysis 

Changes in the protein expression levels of the treated LA-N-1 cells were examined by Western blotting. Briefly, LA-N-1 cells were seeded in a 100 mm dish (2.5 × 10^6^ cells/dish) and incubated with either solvent control (0.5% ethanol) or various concentrations of DHA or EPA for 48 hours. After incubation, the cells were trypsinized, washed and the total proteins were extracted by cold cell lysis buffer (0.03% digitonin in PBS, w/v). The protein concentrations were determined by Bradford reagent. SDS-PAGE was performed to resolve the protein samples and 10% polyacrylamide gel was chosen based on the size of the target proteins. After electrophoresis, the gel was transferred to a PVDF membrane and was tagged with the following primary antibodies: mouse anti-human Bax, Bcl-X_L,_ caspase-3, caspase-8 and CDK2 antibodies (Santa Cruz Biotechnology, Dallas, Texas, USA), mouse anti-human β-actin antibody (Sigma-Aldrich Co. St. Louis, MO, USA), and rabbit anti-human caspase-9 and cyclin E antibodies (Santa Cruz Biotechnology, Dallas, Texas, USA). The membrane was then incubated with HRP-conjugated secondary antibodies (GE Healthcare Limited, Buckinghamshire, UK) and finally developed with ECL reagent (Santa Cruz Biotechnology, Dallas, Texas, USA).

### 2.10. Statistical Analysis 

Experiments were repeated at least three times and only the results of the most representative experiments are shown. The data are presented as the arithmetic mean ± standard deviation (SD). One-way analysis of variance (ANOVA) was used to determine the significant difference between the n-3 PUFA-treated group and the control group. The differences were considered as statistically significant at *p* < 0.05.

## 3. Results

### 3.1. DHA and EPA Inhibit the In Vitro Growth of Human Neuroblastoma LA-N-1 Cells 

**Figure 1:**
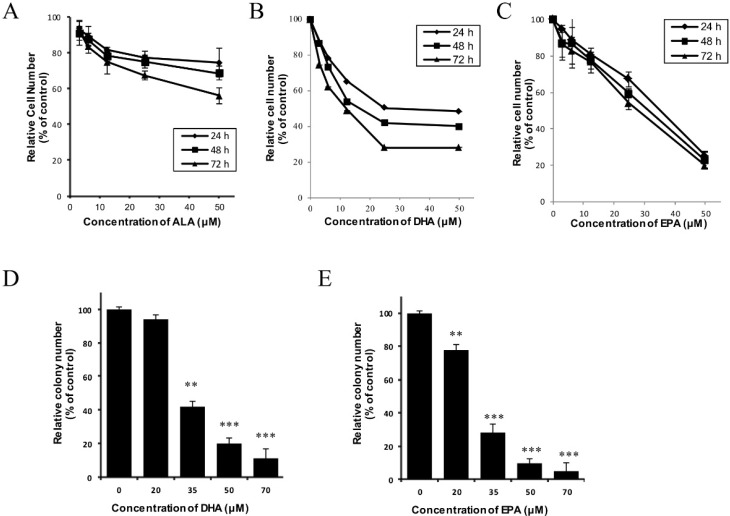
Growth-inhibitory effects of ALA, DHA and EPA on the human neuroblastoma LA-N-1 cells. (**A**–**C**) LA-N-1 cells were incubated with various concentrations of ALA (**A**), DHA (**B**) or EPA (**C**) for 24 hours, 48 hours or 72 hours. Cells treated with 0.5% ethanol acted as the control. After incubation, the viability and metabolic activity of cells were determined by MTT assay. The results are expressed as relative cell number (% of control) ± SD of quadruplicate measurements; (**D**,**E**) LA-N-1 cells were incubated with various concentrations of DHA (**D**) or EPA (**E**) for 24 hours. Cells treated with 0.5% ethanol acted as the control. After incubation, conditioned medium was replaced by complete RPMI medium every 3 days. The colonies were fixed with methanol, stained with Hemacolor staining solutions and counted under light microscopy after 6 days. The results are expressed as relative colony number (% of control) ± SD. ** *p* < 0.01; *** *p* < 0.001.

The growth-inhibitory effect of three common naturally-occurring omega-3 polyunsaturated fatty acids, ALA, DHA and EPA, on the human neuroblastoma LA-N-1 cells was examined by the MTT assay. As shown in Figure 1A–C, all three n-3 PUFAs exerted an anti-proliferative effect towards LA-N-1 cells in a time- and concentration-dependent manner. The approximate 50% inhibitory concentrations (IC_50_) of DHA and EPA on LA-N-1 cells at 48 hours treatment were found to be 18 ± 1 μM and 35 ± 2 μM, respectively, whereas the IC_50_ for ALA on LA-N-1 cells at 48 hours treatment was found to be >50 μM. Due to the relatively higher potency of DHA and EPA with respect to their anti-proliferative effect on LA-N-1 cells, they were chosen to be the specific targets for further mechanistic studies. The growth-inhibitory effect of DHA and EPA on LA-N-1 cells was confirmed by using the colony formation assay (Figure 1D–E). Remarkably, DHA and EPA exhibited minimal, if any, direct cytotoxicity on normal cells including SD rat primary cortical neuronal cells and murine peritoneal macrophages, and non-tumorigenic human cell lines including the human embryonic kidney HEK-293 cells and human hepatocyte-like WRL-68 cells, as the percentage viability remained >80% when these cells were incubated with higher concentrations (up to 100 µM) of DHA or EPA for 72 hours (Figure S1).

### 3.2. DHA and EPA Trigger Cell Cycle Arrest at G_0_/G_1_ Phase and Modulate the Expression Level of Cell Cycle-Regulatory Proteins 

**Figure 2:**
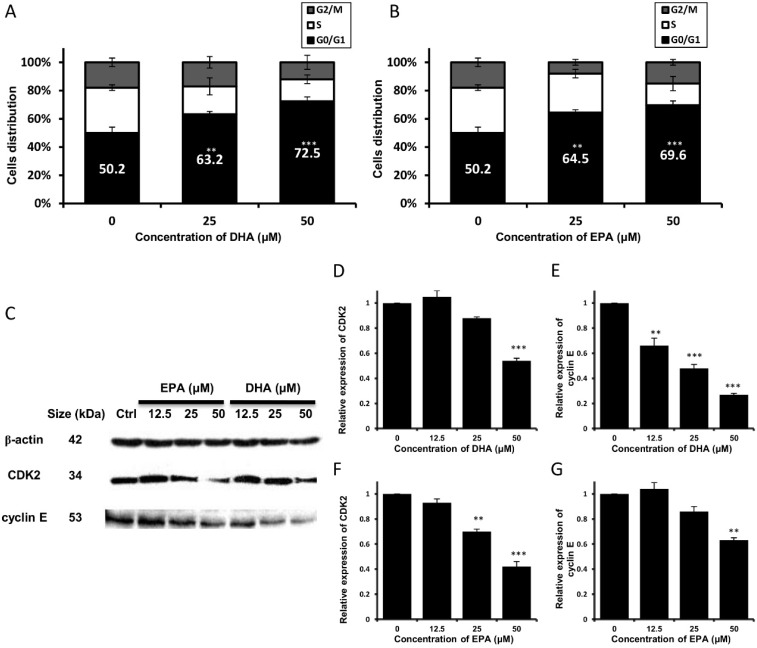
DHA and EPA trigger cell cycle arrest at G_0_/G_1_ phase in LA-N-1 cells and down-regulate the protein expression levels of CDK2 and cyclin E. (**A**, **B**) LA-N-1 cells were treated with various concentrations of DHA (**A**) or EPA (**B**) for 48 hours. Cells treated with ethanol acted as the control. After incubation, cells were stained with PI, and the DNA content was analyzed by the FACSCanto flow cytometer. Cell cycle distribution was analyzed using the Verity’s Modfit LT 3.0 software. Results represent mean ± SD. ** *p* < 0.01; *** *p* < 0.001; (**C**) LA-N-1 cells were incubated with various concentrations of EPA (Lanes 2, 3 and 4) or DHA (Lanes 5, 6 and 7) for 48 hours. Cells treated with ethanol (Lane 1) acted as the control. Protein expression levels of CDK2 and cyclin E were assayed by Western blotting with β-actin served as an internal control. The relative protein expression levels of CDK2 and cyclin E in DHA-treated cells (**D**, **E**) and EPA-treated cells (**F**, **G**) as compared to β-actin were quantified. The results are expressed as relative protein expression levels ± SD. ** *p* < 0.01, *** *p* < 0.001.

To gain a better understanding on the underlying mechanisms for the growth-inhibitory effect of DHA and EPA on human neuroblastoma cells, the cell cycle profiles of DHA- or EPA-treated LA-N-1 cells were examined by flow cytometry. It can be seen that treatment of LA-N-1 cells with DHA (Figure 2A) or EPA (Figure 2B) caused cell cycle arrest at G_0_/G_1_ phase in a concentration-dependent manner, and this was accompanied by a decrease in the percentage of cells at the S phase. Western blotting was then performed to examine the protein expression levels of different cyclin-dependent kinases (CDK) and cyclins, as these proteins promote the progression through different cell cycle checkpoints [25,26]. It was found that the protein expression levels of CDK2 and cyclin E were down-regulated in a concentration-dependent manner after treatment with DHA (Figure 2C–E) and EPA (Figure 2C,F,G), suggesting that the anti-proliferative effect of DHA and EPA might be attributed to their ability to trigger cell cycle arrest of the LA-N-1 cells at the G_0_/G_1_ phase. 

### 3.3. DHA and EPA Induce Apoptosis in LA-N-1 Cells

**Figure 3:**
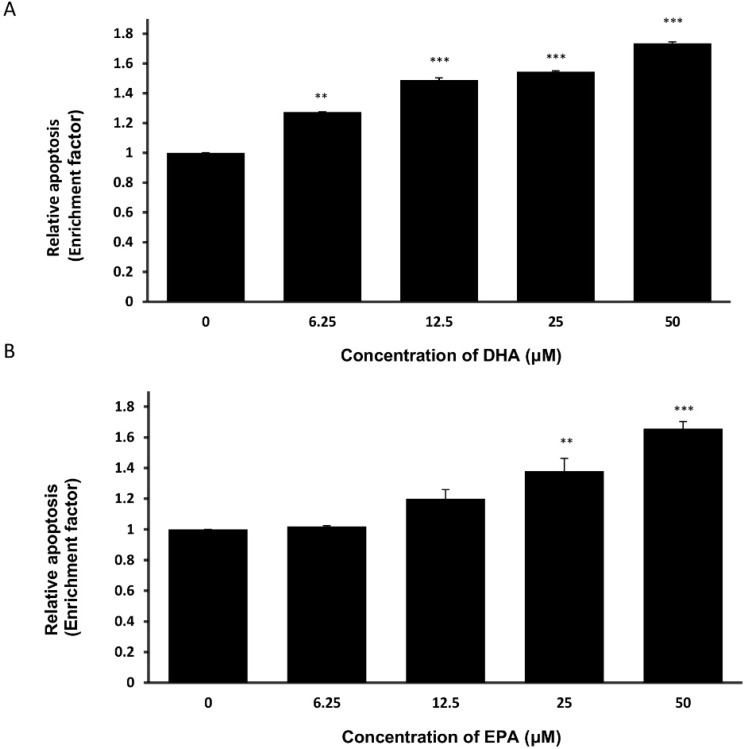
DHA and EPA induce DNA fragmentation in LA-N-1 cells. LA-N-1 cells were treated with various concentrations of DHA (**A**) or EPA (**B**) for 72 hours. Cells treated with ethanol acted as the control. After incubation, the occurrence of DNA fragmentation was detected by the Cell Death Detection ELISA^PLUS^ Kit. The results represent mean enrichment factor ± SD of quadruplicate measurements. ** *p* < 0.01, *** *p* < 0.001.

Apoptosis is a well-regulated process with several key events, one of which is the induction of DNA fragmentation in the cells [27]. To examine whether DHA or EPA could trigger DNA fragmentation in LA-N-1 cells, the Cell Death Detection ELISA^PLUS^ Kit was used according to manufacturer’s instruction. As shown in Figure 3, DHA and EPA could induce DNA fragmentation in LA-N-1 cells in a concentration-dependent manner as reflected by an increase in the enrichment factor. 

**Figure 4:**
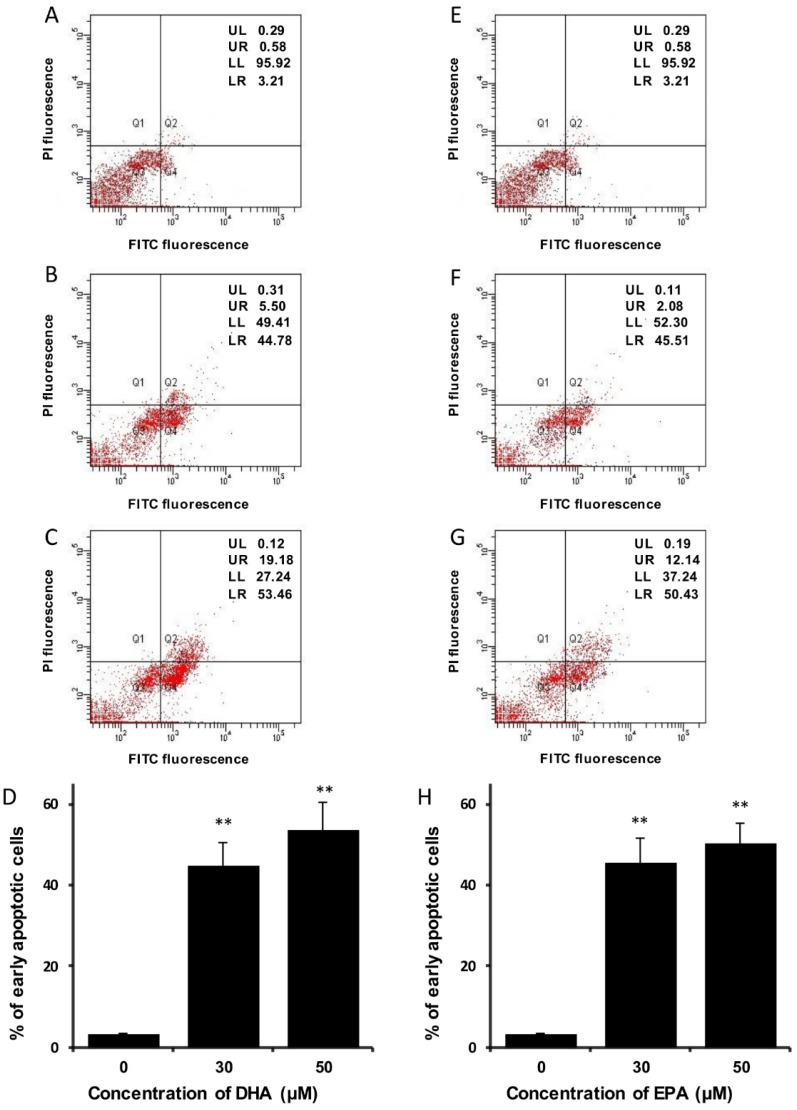
DHA and EPA trigger phosphatidylserine externalization in LA-N-1 cells. (**A**–**D**) LA-N-1 cells were incubated with ethanol control (**A**), 30 or 50 µM DHA (**B**, **C**) for 48 hours. (**E**–**H**) LA-N-1 cells were incubated with ethanol control (**E**), 30 or 50 µM or EPA (**F**, **G**) for 48 hours. After incubation, the cells were stained by Annexin V-GFP fusion protein and PI, and the fluorescence intensity was measured by flow cytometry. The results are quantified and expressed as mean values ± SD (**D**, **H**). ** *p* < 0.01.

**Figure 5:**
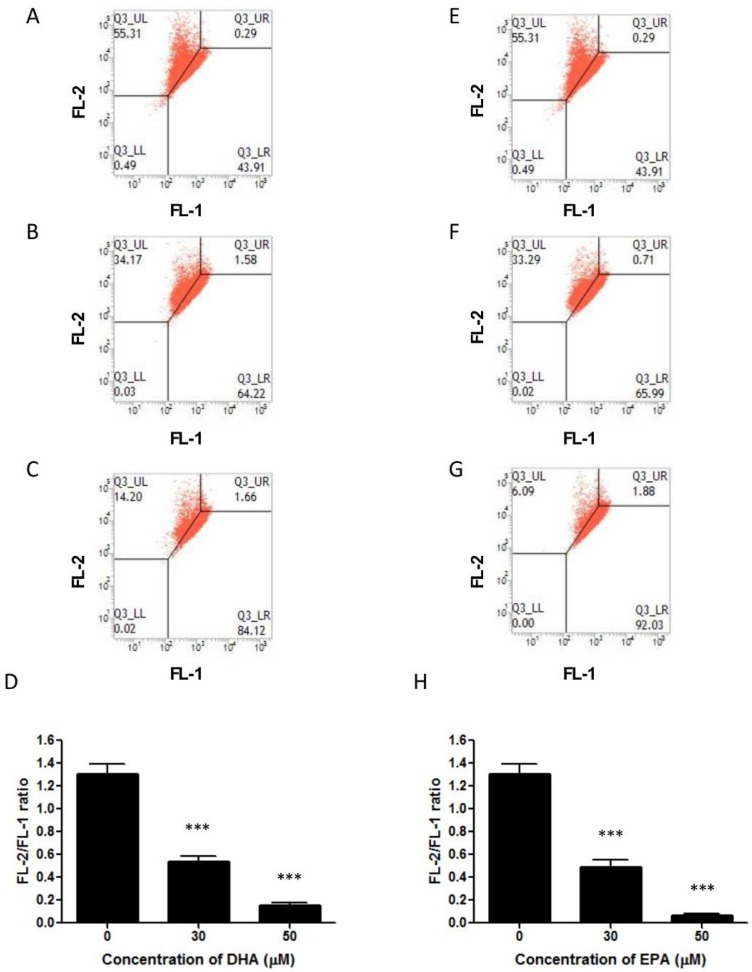
DHA and EPA reduce mitochondrial membrane potential in LA-N-1 cells. (**A**–**D**) LA-N-1 cells were incubated with ethanol control (**A**), 30 or 50 µM DHA (**B**, **C**) for 48 hours. **(E**–**H)** LA-N-1 cells were incubated with ethanol control (**E**), 30 or 50 µM EPA (**F**, **G**) for 48 hours. After incubation, the cells were stained by JC-1 dye and the mitochondrial potentials were detected by flow cytometry. The results are quantified and expressed as mean values ± SD (**D**, **H**). *** *p* < 0.001.

Apart from DNA fragmentation, PS externalization is another hallmark feature of apoptosis [28]. In the present study, Annexin V-GFP fusion protein and PI co-staining method was employed to monitor the PS externalization. Flow cytometric analysis showed that the percentage of cells with PS externalization increased when the cells were treated with DHA (Figure 4A–D) or EPA (Figure 4E–H). In addition, previous studies have suggested that cell cycle arrest might be a result of mitochondrial failure [29], hence the mitochondrial membrane potential of the treated cells was examined by JC-1 dye staining method. It was found that the mitochondrial membrane potential in LA-N-1 cells was reduced after treatment with DHA (Figure 5A–D) and EPA (Figure 5E–H) in a concentration-dependent manner. 

**Figure 6:**
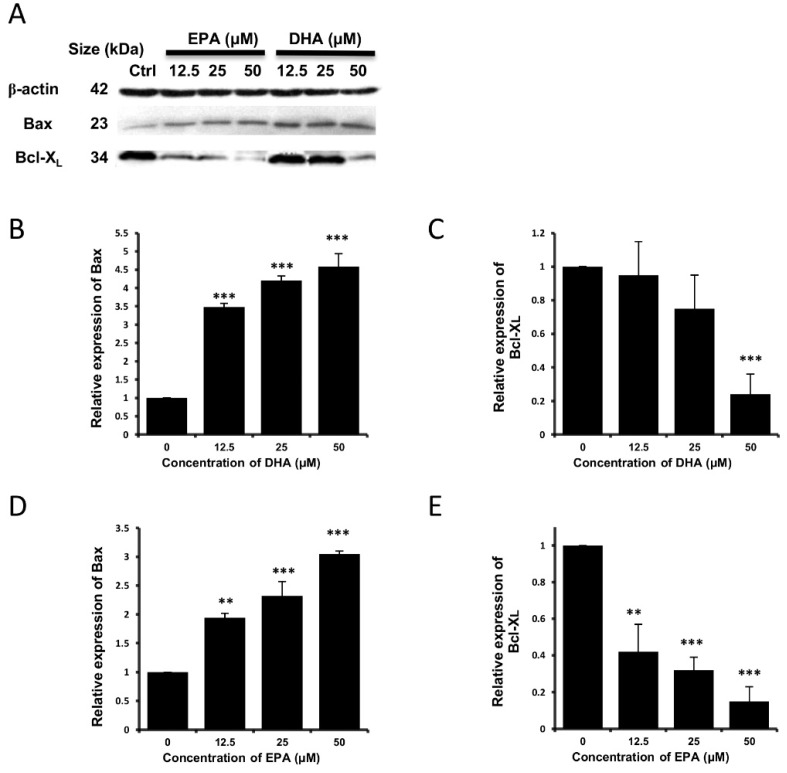
Effects of DHA and EPA on the expression levels of apoptosis-regulatory proteins in LA-N-1 cells. (**A**) LA-N-1 cells were incubated with various concentrations of EPA (Lanes 2, 3 and 4) or DHA (Lanes 5, 6 and 7) for 48 hours. Cells treated with ethanol (Lane 1) acted as the control. The protein expression levels of Bax, Bcl-X_L_ were measured by Western blotting with β-actin serving as an internal control. The relative protein expression levels of Bax and Bcl-X_L_ in DHA-treated cells (**B**, **C**) and EPA-treated cells (**D**, **E**) as compared to β-actin were quantified. The results are expressed as relative protein expression levels ± SD. ** *p* < 0.01, *** *p* < 0.001.

**Figure 7:**
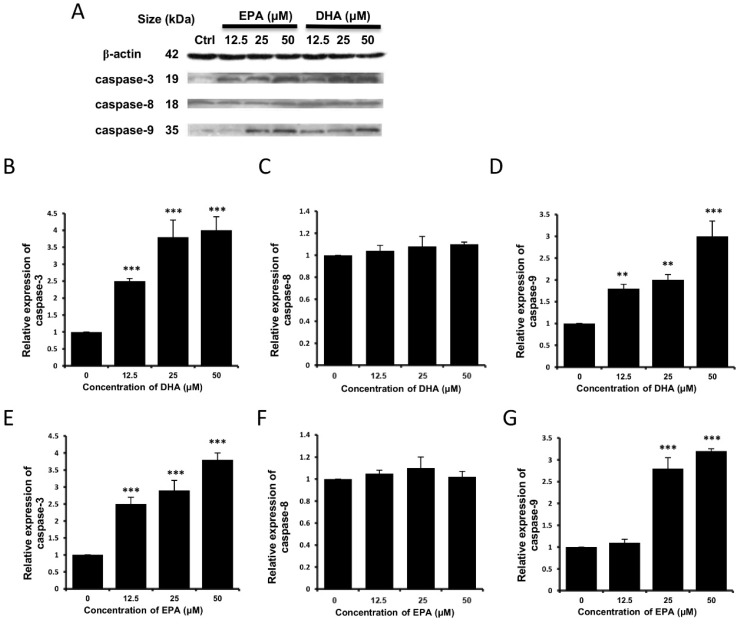
Effects of DHA and EPA on the expression levels of activated caspases in LA-N-1 cells. (**A**) LA-N-1 cells were incubated with various concentrations of EPA (Lanes 2, 3 and 4) or DHA (Lanes 5, 6 and 7) for 48 hours. Cells treated with ethanol (Lane 1) acted as the control. The protein expression levels of activated caspase-3, caspase-8 and caspase-9 were measured by Western blotting with β-actin serving as an internal control. The relative protein expression levels of activated caspase-3, caspase-8 and caspase-9 in DHA-treated cells (**B**–**D**) and EPA-treated cells (**E**–**G**) as compared to β-actin were quantified. The results are expressed as relative protein expression levels ± SD. ** *p* < 0.01, *** *p* < 0.001.

Apoptosis can be triggered by both intrinsic and extrinsic pathways, and the mitochondrial dysfunction is the major characteristic of the intrinsic pathway [30]. To unravel the molecular mechanisms of the occurrence of apoptosis, the protein expression levels of several apoptosis-regulatory proteins and different caspase proteins were examined by Western blot analysis. It could be seen that the expression level of the pro-apoptotic Bax protein was increased, accompanied by a decrease in the expression level of the anti-apoptotic Bcl-X_L_ protein in a concentration-dependent manner in both the DHA-treated (Figure 6A–C) or EPA-treated cells (Figure 6A,D,E). It was reported that Bax and Bcl-X_L_ in the Bcl-2 family are responsible to elicit the intrinsic pathway of apoptosis [31]. Furthermore, our results suggested that the protein expression levels of activated caspase-3 (Figure 7A,B,E) and caspase-9 (Figure 7A,D,G) increased after treatment of DHA or EPA, whereas the expression level of activated caspase-8 protein did not alter significantly (Figure 7A,C,F). Collectively, our results suggest that the occurrence of apoptosis triggered by DHA and EPA on LA-N-1 cells might be mediated via the intrinsic pathway but not the extrinsic pathway.

## 4. Discussion

The LA-N-1 cell line is a well-established human neuroblastoma cell line with *N-myc* gene amplification [22,32], which is found to be related with the invasiveness and severity of neuroblastoma [33]. LA-N-1 cells have been commonly used as an *in vitro* model for the study of anti-tumor effects through various mechanisms, such as triggering cell cycle arrest, inducing apoptosis and differentiation, or reducing *N-myc* expression [24,34,35]. In the present study, it was found that DHA and EPA, two common naturally-occurring n-3 PUFAs, could significantly inhibit the growth of the human neuroblastoma LA-N-1 cells in a time- and concentration-dependent manner, suggesting that n-3 PUFAs might be effective in suppressing the growth of aggressive neuroblastoma. The results are consistent with previous reports which demonstrated the anti-tumor effects of n-3 PUFAs on various cancer cell lines [36,37,38]. On the other hand, it was found that ALA was less potent than DHA or EPA with regard to its anti-proliferative effect on human neuroblastoma cells, which was in line with previous findings [38,39]. Several reports have suggested that n-3 PUFAs could exert their anti-tumor effects in different cancer cells through triggering cell cycle arrest [17,40]. To examine whether DHA and EPA inhibited the growth of LA-N-1 cells by means of cell cycle arrest, the cell cycle profiles of the treated cells were examined by PI staining. Flow cytometric analysis suggested that DHA and EPA triggered cell cycle arrest at the G_0_/G_1_ phase in a concentration-dependent manner, and accompanied by a decrease in the percentage of cells at the S phase. CDK and cyclins are thought to be involved in the regulation of cell cycle progression, and G_0_/G_1_ phase is regulated by CDK2/4/6, cyclin D1/D2/D3 and cyclin E [25]. In the present study, we found that there was a decrease in the protein expression levels of CDK2 and cyclin E in the DHA- or EPA-treated LA-N-1 cells, indicating that the growth-inhibitory effect of DHA and EPA on LA-N-1 cells might be due to the triggering of cell cycle arrest at G_0_/G_1_ phase.

In addition to triggering cell cycle arrest, induction of apoptosis in the cancer cells is another mechanism that might account for the anti-proliferative effect of DHA and EPA as described previously [37,41]. Using the Cell Death Detection ELISA^PLUS^ Kit, it was found that DHA and EPA could induce DNA fragmentation in LA-N-1 cells. In addition, other hallmarks of apoptosis, such as PS externalization and mitochondrial membrane depolarization, were examined in the treated cells. After the treatment of DHA or EPA, there was an increase in the percentage of cells with PS externalization and mitochondrial membrane depolarization, these findings further supported that DHA and EPA could elicit apoptosis in LA-N-1 cells. Bcl-2 family is a group of proteins that regulate the mitochondrial membrane permeabilization and the induction of apoptosis [42]. Western blot analysis indicated that the protein expression level of the pro-apoptotic Bax protein was increased, and this was accompanied by a decrease in the expression level of anti-apoptotic Bcl-X_L_ protein in the DHA- or EPA-treated LA-N-1 cells. Similarly, it was shown that both DHA and EPA could reduce expression of the anti-apoptotic proteins Bcl-2 and Bcl-X_L_ in cultured breast cancer MDA MB-231 cells [19,43]. It is well known that apoptosis is caspases-dependent and can be triggered through the intrinsic and extrinsic pathways. Activation of intrinsic pathway is the result of mitochondrial dysfunction and is characterized by an increase in the expression levels of the activated caspase-3 and caspase-9 proteins. On the other hand, the extrinsic pathway is characterized by an increase in the activated caspase-8 and caspase-10 protein expression levels [44]. Our findings showed that DHA and EPA could up-regulate the expression levels of the activated caspase-3 and caspase-9 proteins, while the expression level of activated caspase-8 protein remained unchanged, suggesting that the induction of apoptosis in LA-N-1 cells after treatment with DHA and EPA might possibly due to the activation of the intrinsic pathway, but not the extrinsic pathway.

## 5. Conclusions

To conclude, DHA and EPA are found to exhibit growth-inhibitory effect on the human neuroblastoma LA-N-1 cells by triggering cell cycle arrest at the G_0_/G_1_ phase and inducing apoptosis through activation of the intrinsic pathway. Notably, we found that DHA and EPA exerted little, if any, direct cytotoxicity on the normal murine and rat cells, as well as on non-tumorigenic human cell lines. Since n-3 PUFAs can be easily found in our daily diet and exert numerous beneficial effects on our body health, DHA and EPA are potential anti-cancer agents, which might be used for the adjuvant therapy or combination therapy with the conventional anti-cancer drugs for the treatment of some forms of human neuroblastoma with minimal toxicity and fewer side effects. It would be of interest to know whether the combinations of DHA or EPA with the conventional anti-cancer agents might be synergistic in terms of their anti-tumor effect. 

## Supplementary Files

Supplementary File 1
