# Supplementary material for: Omega-3 Polyunsaturated Fatty Acids Trigger Cell Cycle Arrest and Induce Apoptosis in Human Neuroblastoma LA-N-1 Cells
Source: Nutrients. 2015 Aug 18;7(8):6956–73. doi: 10.3390/nu7085319 (PMC4555158; doi:10.3390/nu7085319)
Supplement: Supplementary File 1 [file nutrients-07-05319-s001.docx]

Supplementary Information


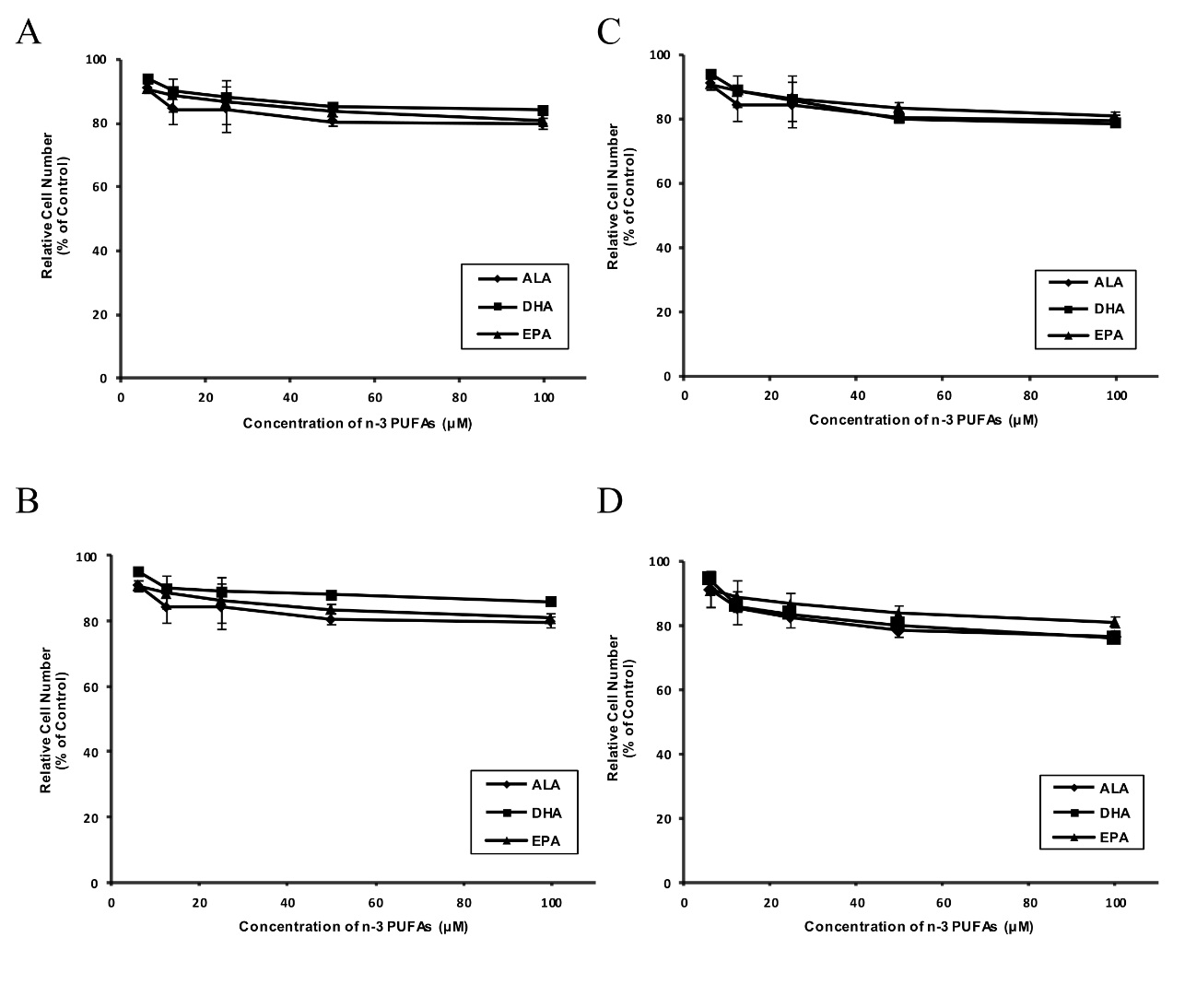


**Figure S1.** Effect of n-3 PUFAs on normal or non-tumorigenic cells. (**A**) Murine peritoneal macrophages (3 × 10^5^ cells/well); (**B**) SD Rat primary cortical neurons (2 × 10^5^ cells/well); (**C**) human embryonic kidney HEK-293 cells (8 × 10^3^ cells/well) or (**D**) human hepatocyte-like WRL-68 cells (5 × 10^3^ cells/well) were seeded in 96-well plates and then treated with solvent control (0.5% ethanol) or various concentrations of n-3 PUFAs (ALA, DHA or EPA). After incubation for 72 h, the viability and metabolic activity of the cells were determined by the MTT assay. The results were expressed as relative cell number (% of control) ± SD of quadruplicate measurements.

© 2015 by the authors; licensee MDPI, Basel, Switzerland. This article is an open access article distributed under the terms and conditions of the Creative Commons Attribution license (http://creativecommons.org/licenses/by/4.0/).
